# Case report of atypical presentation of Bartter’s syndrome: hypertension and Hypokalemic nephropathy in a female patient

**DOI:** 10.1093/omcr/omae215

**Published:** 2025-03-28

**Authors:** Emad A Odeh, Jaber H Jaradat, Raghad Amro, Seif Jankhout, Abdulqadir J Nashwan

**Affiliations:** Department of Internal Medicine, Nephrology Department, Al-Basheer Hospital, Jordanian Ministry of health, Jabal al-Ashrafiyeh, At Taj Street, Amman Qasabah District, Amman 11151, Jordan; Faculty of Medicine, Mutah University, Karak Qasabah District, Mu'tah St, Al Karak 61710, Jordan; Faculty of Medicine, Mutah University, Karak Qasabah District, Mu'tah St, Al Karak 61710, Jordan; Faculty of Medicine, Mutah University, Karak Qasabah District, Mu'tah St, Al Karak 61710, Jordan; Nursing Department, Hamad Medical Corporation, Hamad Medical City, Al Rayyan Road, Doha 3050, Qatar

**Keywords:** Bartter’s syndrome, hypokalemic nephropathy, hypertension, electrolyte imbalance, case report

## Abstract

Bartter’s syndrome (BS) is a rare hereditary disorder characterized by tubulopathies that affect salt reabsorption in the thick ascending limb of the Henle’s loop. While BS typically presents with electrolyte imbalances and normal blood pressure, atypical presentations can complicate its diagnosis and management. We report the case of a 21-year-old female with BS diagnosed clinically at birth who presented with cough, vomiting, and elevated blood pressure. Despite a positive family history of BS, her presentation was unique with hypertension and hypokalemic nephropathy. Diagnostic evaluations during hospital admission revealed electrolyte imbalances, kidney dysfunction, and renal histopathological findings that were consistent with hypokalemic nephropathy. This case highlights the diverse clinical manifestations and potential complications of BS and emphasizes the importance of thorough evaluation and multidisciplinary management. Further research is needed to elucidate the underlying mechanisms linking BS with hypertension and hypokalemic nephropathy, and to guide targeted therapeutic strategies for complex presentations.

## Introduction

Bartter’s syndrome (BS), first described by Frederic Bartter in 1962, represents a diverse collection of tubulopathies marked by both recessive and dominant autosomal inheritance. This syndrome arises from dysfunction in salt reabsorption in the thick ascending limb of Henle’s loop [1]. BS is a rare condition occurring in approximately 1 in 1 000 000 individuals with an equal male-to-female ratio and normal life expectancy [2]. These genetic variations are key factors that influence the diverse symptoms and clinical features observed in individuals with Bartter syndrome [3].

Clinical presentation of BS include polyuria, polydipsia, volume contraction, muscle weakness, growth retardation, and occasional nephrocalcinosis with significant electrolyte imbalance such as hypokalemia and hypochloremia, and to a lesser extent hypercalcemia, hyperuricemia, and hypomagnesemia [4].

To our knowledge, this is the first case of Bartter’s syndrome presenting with hypokalemic nephropathy and hypertension. Moreover, this case is considered the 20^th^ case to be reported about hypokalemic nephropathy in general [5].

## Case presentation

A 21-year-old female patient with Bartter’s syndrome diagnosed clinically at birth presented to the hospital with complaints of cough and vomiting. On physical examination, she had elevated blood pressure (190/135 mm Hg). She confirmed the absence of any other symptoms associated with elevated blood pressure such as headache, dizziness, chest pain, changes in vision, or shortness of breath. Her blood pressure was controlled and managed with nifedipine 20 mg and furosemide 40 mg before referring the patient to a central hospital for further investigation. She regularly takes folic acid, ferrous gluconate, and potassium gluconate and has no known allergies.

**Figure 1:**
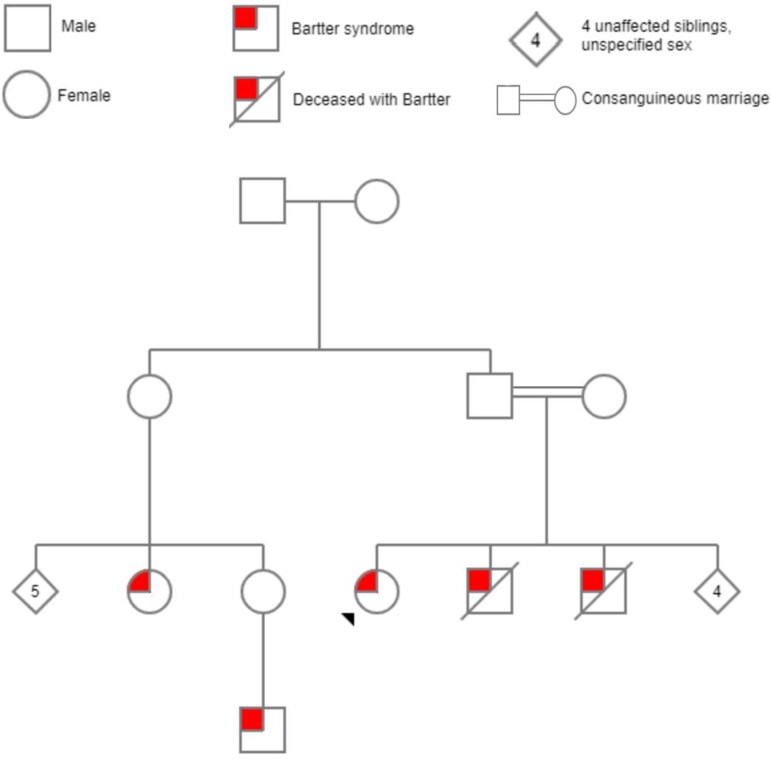
Shows the family pedigree of the patient.

She had a positive family history of Bartter’s syndrome, two brothers, a cousin, and the son of the cousin. While her brothers died at the age of 15-years-old of chronic kidney disease (CKD), her cousin and her other cousin’s son are still alive and well (Fig. 1).

On physical examination, the patient was conscious, oriented, and non-distressed, with a heart rate of 153 beats per minute, and was afebrile with a normal body temperature, and the remainder was insignificant.

During the weeklong admission period, the patient underwent extensive diagnostic evaluations to investigate the underlying cause of her elevated blood pressure. Daily laboratory testing are presented in Table 1, which includes all the abnormal blood and urine test results during the first and the second admission. The CBC test revealed low RBC counts, PCV, basophilia, and eosinophilia and high MCH, MCHC, and RDW. Serum electrolyte levels remained mostly within the normal range, although serum potassium levels decreased transiently (2.97 mmol/l) during admission; however, a month later, they returned to normal. Kidney function tests showed elevated serum creatinine levels over the past two years, with baseline readings ranging from 98 μmol/l to 122 μmol/l; however, other parameters were within normal limits. Moreover, the urine analysis was consistently normal throughout admission.

**Table 1:** Shows laboratory values of the patient on the two admissions of blood and urine samples.

**Blood and urine tests**	**Values on 1** ^**st**^ **admission**	**Values on 2** ^**nd**^ **admission**	**Normal ranges**
**RBC count (10** ^**6**^ **/ul)**	2.99	4.11	4.3–5.1
**PCV (%)**	26.9	35.8	35–45
**MCH (PG)**	41.8	30.2	27–34
**MCHC (g/dl)**	46.5	34.6	32–36
**RDW-CV %**	15.1	13.2	11.6–14.6
**Eosinophils %**	4.5	4.2	0–4
**Basophils count (10** ^**3**^ **/ul)**	0.03	0.05	0–0.01
**Basophils %**	0.5	0.8	0.01–0.1
**Serum creatinine (umol/l)**	113.6	118	44–80
**Urine RBC**	4–6	12–14	
**Urine calcium (mmol/l)**	-	0.09	1.7–5.3
**Urine magnesium (mmol/l)**	-	0.42	3–5
**Urine creatinine (umol/l)**	-	2214	2550–20 000
**Urine sodium (mmol/l)**	-	11	45–100
**Plasma renin activity (ng/l)**	-	16	7.54–63.3

RBC, Red Blood Cells; PCV, Packed Cell Volume; MCH, Mean Corpuscular Hemoglobin; MCHC, Mean Corpuscular Hemoglobin Concentration; RDW-CV, Red Cell Distribution Width-Coefficient of Variation

Multiple imaging investigations were performed, including brain CT, chest radiography, and renal ultrasonography, and all these imaging investigations were reassuring with no visible abnormalities.

During her first admission, the patient received antihypertensive medications, including Nifedipine, Indapamide, Candesartan, and Captopril, in addition to the drugs/supplements she was actively taking before admission, which effectively managed her elevated blood pressure. She was discharged with stable vital signs and was prescribed spironolactone 25 mg once daily, with instructions for follow-up visits to the nephrology clinic and blood pressure monitoring.

However, three weeks later, the patient again presented with elevated blood pressure (158/106 mmHg), prompting a second admission for further evaluation. Renal biopsy and additional laboratory tests including immunological, serum, and urine analyses were performed.

The patient’s C3 and C4 complement levels were within the normal range, but the serum ANA was positive with a 1/80 titer and a speckled pattern. Regarding electrolytes in urine, calcium, magnesium, creatinine, and sodium levels were low, but the potassium level was normal. Several urine analysis tests performed during the second admission after renal biopsy revealed red RBCs in the urine. Finally, the patient’s renin test results were normal.

**Figure 2:**
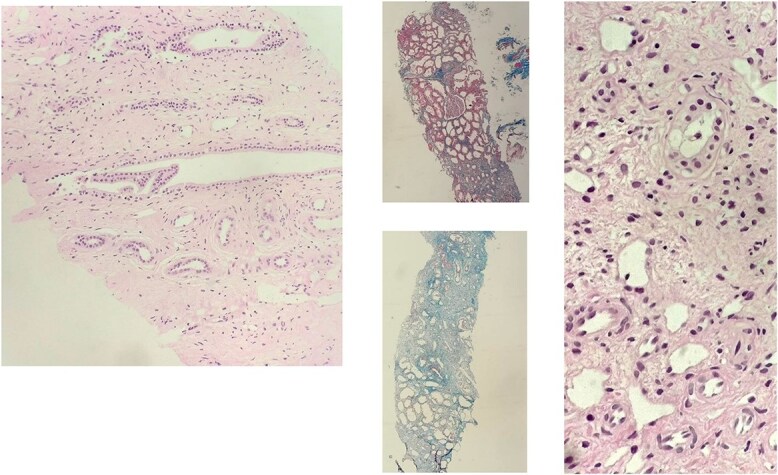
Shows images from the renal biopsy showing interstitial fibrosis, tubular atrophy, and sclerosed glomeruli. One glomerulus shows segmental sclerosis, another one is unremarkable. Interstitial fibrosis and tubular atrophy is seen in about 70% and inflammatory cells are predominantly composed of lymphocytes mainly and rarely eosinophils. Some of the tubules are dilated with degenerated changes and occasional intracytoplasmic vacuoles and hyalin casts are also observed. Some of the blood vessels are hyperplastic.

The surgical pathological report of the patient’s renal biopsy showed three glomeruli: one glomerulus appeared unremarkable, another displayed segmental sclerosis, and the third was globally sclerosed. The tubulointerstitial compartment showed 70% interstitial fibrosis and tubular atrophy with foci of chronic inflammatory cell infiltrates predominantly composed of lymphocytes with rare eosinophils, some of which were dilated with degenerative changes, and hyaline casts were observed. These tubulointerstitial changes and laboratory test findings were compatible with hypokalemic nephropathy. The blood vessels were thickened and some showed hyperplastic arteriosclerosis (Fig. 2). The patient was discharged after stabilization of her blood pressure, advised to monitor her blood pressure daily, and had follow-up visits to the nephrology clinic.

## Discussion

Bartter’s syndrome is a rare autosomal recessive disorder characterized by hypokalemia, hypochloremia, metabolic alkalosis, and hyperaldosteronism. This syndrome differ from typical presentation in patients with hyperaldosteronism in which they are younger (children and young adults), with normal blood pressure, and had growth retardation [6–8].

Clinical suspicion should be raised when children and young patients have hypokalemic metabolic alkalosis with normal blood pressure. However, in adults, these manifestations should prompt the exclusion of diuretic use and other possible causes, as they are rare in adults [6].

The diagnosis of BS in our patient followed the typical clinical presentation of BS [6, 8], as she was diagnosed after birth and followed up for up to now. Furthermore, our patient was presented several times as a case of hypertension.

One notable aspect of this case was the association of BS with hypertension and glomerulonephritis, which has rarely been reported in the literature. Hypertension is not a typical feature of BS, and when present, it often raises the suspicion of an alternative diagnosis or secondary cause. In our patient, hypertension was managed with antihypertensive medications including Nifedipine, Indapamide, Candesartan, and Captopril, along with the existing treatment regimen for BS.

The presence of glomerulonephritis associated with BS adds to the complexity of the clinical picture. Despite the typical presentation at the time of BS diagnosis, developing hypertension and presenting emergently as a case of malignant hypertension (HTN) was vague and against the cardinal feature of BS (normal blood pressure). Chan et al. and Pejovic et al. described preterm infants diagnosed with BS and hypertension. [9, 10]. Another case of BS was diagnosed in the context of hypertension [11]. The diagnosis of BS with HTN is rare; however, it has been reported in the literature. The BP in our patient was normal, suggesting another cause of hypertension.

Hypokalemia is a common clinical feature of BS; however, hypokalemic nephropathy, as in our patient, is a rare complication. Hypokalemic nephropathy is a tubulointerstitial disease caused by prolonged hypokalemia, metabolic alkalosis, and progressive loss of renal function [12].

The histopathological study of our case revealed the usual characteristics of hypokalemic nephropathy like interstitial fibrosis, tubular atrophy, and chronic inflammatory cell infiltration [13].

Another rare presentation of our patient was positive serum ANA with a speckled pattern, which raises the possibility of an underlying autoimmune disorder contributing to the patient status. Although autoimmune diseases are not commonly associated with BS, the presence of positive ANA warrants consideration and further investigation to elucidate potential autoimmune-mediated renal involvement. Nasir et al., described a rare diagnosis of BS in an adult suspected to be caused by an underlying autoimmune mechanism as the patient was SCLC 70 positive in the absence of other systemic manifestations [14]. Susceptibility to an underlying autoimmune disorder contributed to the complexity of our case. The most common autoimmune disorder was Sjogren’s syndrome [14].

In conclusion, this case highlights the diverse clinical manifestations and potential complications of Bartter’s syndrome, emphasizing the importance of thorough evaluation and multidisciplinary management to optimize patient care. A lesson from our case is to do kidney biopsy as soon as possible in patients with Bartter’s syndrome presenting with hypertension and hypokalemia, to protect the undamaged part of the kidneys from suspect underlying hypokalemic nephropathy. Further research is needed to better understand the underlying mechanisms linking BS with hypertension and glomerulonephritis and to guide the development of targeted therapeutic strategies for such complex presentations.

## Data Availability

The data presented in this study are available within the article.
